# Identification of Potential Vectors and Species Density of Tsetse Fly, Prevalence, and Genetic Diversity of Drug-Resistant Trypanosomes in Kenya

**DOI:** 10.3390/pathogens14121207

**Published:** 2025-11-26

**Authors:** Ivy S. Okello, Samuel G. Onyoyo, Isaiah N. Kiteto, Sylvia M. Korir, Seth. O. Onyango

**Affiliations:** 1 Kenya Tsetse and Trypanosomiasis Eradication Council, Nairobi P.O. Box 66290-00800, Kenya; 2 Biotechnology Research Institute, Kenya Agricultural and Livestock Research Organization, Kikuyu P.O. Box 362, Kenya

**Keywords:** tsetse flies, trypanosomes, apparent density, genetic diversity, drug resistance

## Abstract

Tsetse flies are major vectors of trypanosomes in Sub-Saharan Africa, posing risks to livestock and human health. This study investigated the diversity, distribution, and infection rates of tsetse species, as well as the genetic diversity of drug resistance-associated trypanosome strains in Kenya. Flies were collected from Kwale, Taita-Taveta, Kajiado, Narok, and Turkana counties between November 2024 and February 2025. DNA analyses targeting rRNA and transporter genes (*TbAT/P2*, *E6M6*, *DMT*, *TcoAde2*) identified infections and resistance-associated mutations among 4693 sampled flies. Apparent density was highest in Kwale (101.52 flies/trap/day) and lowest in Turkana (1.18). Species distribution varied by county, with Kwale dominated by *G. pallidipes*, *G. austeni*, and *G. brevipalpis*; Taita-Taveta *G. pallidipes*, and *G. brevipalpis*; Kajiado *G. pallidipes* and *G. longipennis*; Narok *G. pallidipes*, *G. brevipalpis*, *G. swynnertoni*, and *G. longipennis*; and Turkana only *G. pallidipes*. *Trypanosoma congolense* was most prevalent, especially in Kwale, while *T. brucei* was common in Kajiado and Kwale. *G. brevipalpis*, *G. austeni*, and *G. pallidipes* showed higher infection risks. Drug resistance-associated *T. congolense* strains were found in Kwale and Taita-Taveta, with *TcoAde2* and *E6M6* gene diversity linked to Kenyan isolates. These findings highlight the need for targeted control of high-risk tsetse species and drug-resistant trypanosomes in Kenya.

## 1. Introduction

Tsetse flies (*Glossina* spp.) are known to be the main vectors transmitting trypanosomes to humans and livestock. In livestock, the disease is referred to as African animal trypanosomiasis (AAT). AAT tremendously affects livestock production in Sub-Saharan African countries, negatively influencing food security [1,2,3]. Different species of trypanosomes have been reported to be transmitted by tsetse flies to domesticated livestock. These include *T. congolense*, *T. brucei*, *T. vivax*, *T. godfreyi*, *T. evansi*, and *T. simiae* [4]. Other hexapod vectors known to transmit the animal trypanosomes include the *Stomoxys* and Tabanids that mechanically transmit *T. evansi*, *T. vivax*, and *T. theileri* trypanosome species [5,6,7]. The prevalence of different species of tsetse flies and their infection rates has been reported in a recent review study in eastern and western Sub-Saharan Africa regions [8]. In East Africa, *G. pallidipes*, followed by *G. fuscipes* and *G. swynnertoni*, were identified as the most common species and those causing most infections in descending order [8]. On the other hand, in West Africa, *G. palpalis* and *G. mortisans* were detected as the most common groups of tsetse flies causing infections. Studies have been conducted to identify potential vectors of Rift Valley fever virus (RVF) in mosquitoes from Rwanda, and the mosquito species population distribution and abundance [9]. In Kenya, a recent study carried out by Kenya Tsetse and Trypanosomiasis Eradication Council (KENTTEC) on a national atlas aimed at controlling tsetse-transmitted AAT identified eight different species of tsetse flies found in five different regions. However, this study did not include the detection of trypanosome species in the different tsetse species from the different regions of Kenya [1]. In addition, this study used a microscopy method of detection of trypanosome infections in domesticated livestock, which is considered less sensitive compared to the molecular PCR method. Studies have been conducted on the genetic diversity of trypanosomes infesting cattle from Côte d’Ivoire [10]. In Kenya, studies have been conducted on the seasonal variation in tsetse fly density and infection rates and the prevalence of trypanosomes associated with drug resistance in tsetse flies from certain endemic regions [11,12]. However, no known studies have focused on detecting the genetic diversity of trypanosomes infesting different tsetse fly species from different endemic regions in Kenya.

Chemoresistance to trypanocides has been a significant problem in Sub-Saharan African countries, slowing the progress of AAT elimination [8,13]. Mutations in various transporter genes—such as *TbAT1*, *DMT*, *E6M61*, and *TcoAT1*—which mediate the uptake of trypanocidal drugs like isometamidium and diminazene, have been linked to the development of drug-resistant trypanosomes [12,14,15,16,17,18]. Restriction fragment length polymorphism (RFLP) analysis has been utilized to detect *T. congolense* species linked to drug resistance [16,18,19], while multiple studies in Kenya have explored the prevalence of resistant trypanosome strains [20,21]. However, very limited studies have focused on determining the prevalence of the trypanosome associated with drug resistance in tsetse flies from different endemic regions. Therefore, identifying potential trypanosome vectors, their apparent density and distribution, and the drug resistance status of trypanosomes is essential for determining regions with high abundance of different tsetse fly species. This information can be used for further studies, evaluating the transmission risk of trypanosomes to humans and animals, and guiding more effective prevention and control strategies for African trypanosomiasis, as well as forecasting AAT disease outbreaks. The purpose of this study, hence, was to determine the tsetse fly species that may serve as vectors of trypanosomes, and determine their apparent density, distribution, infection, and drug resistance status of trypanosomes in five counties of Kenya.

## 2. Materials and Methods

### 2.1. Trapping of Tsetse Flies

**Figure 1:**
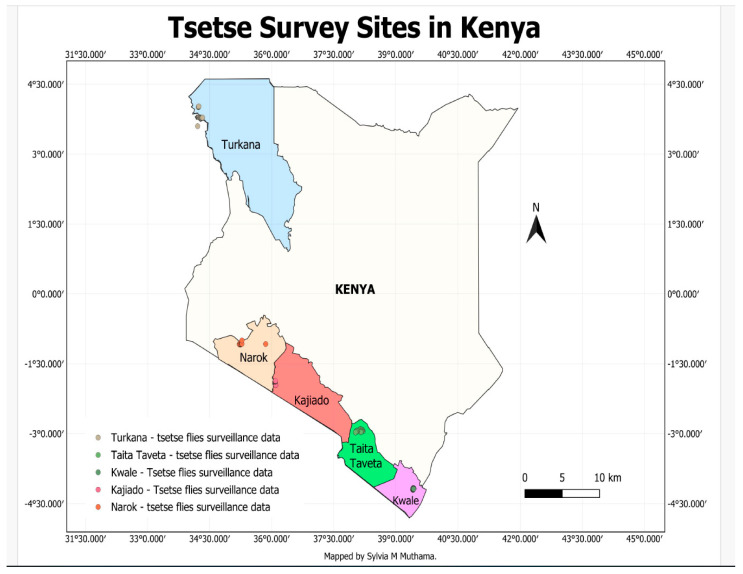
A map showing respective regions where tsetse survey was conducted in Kenya.

Tsetse flies were trapped during baseline survey and monitoring activities conducted by KENTTEC in the five counties. Trapping was carried out over three consecutive days in each county. The geographical coordinates of all trapping sites were geo-referenced and recorded using GPS. A map was then drawn using the Quantum Geographical Information System (QGIS). A total of 25 assorted traps, including NZI, NGU, and Biconical traps, were deployed in the Kwale, Taita-Taveta, Kajiado, Narok, and Turkana counties of Kenya in different transects—Figure 1. Narok County traps were deployed at Enonkishu conservancy in block 12 wetland, block 13 forest, Mara training center and block 7 and 9; Kajiado County traps were deployed along OI Kiramatian Conservancy–Shompole Conservancy in the Nguruman area; Kwale County traps were deployed along the Marere Springs–Marere Bridge in the Shimba Hills national reserve, and Godoni Airstrip–Mwadabara pump station II in the Shimba Hills national reserve; Turkana County traps were deployed in a transect between Nawuontos and Oropoi, while Taita-Taveta traps were deployed at Rhino Valley and Five Sisters Hills–Mzima springs in Tsavo West national reserve. These traps were sourced from KENTTEC and placed in different transects and separated by not more than 200 m within each county. Prior treatment of the panels with phenol and acetone was performed to lure the flies. Phenol sachets were inserted into pockets sewn into the trap-target cloth, while acetone bottles were affixed to the central pole of each trap target. Release rates were approximately 0.4 mg/h for 4-methylphenol, 0.02 mg/h for 3-n-propylphenol, and 500 mg/h for acetone; these rates depended on the polyethylene sachets’ surface area for the phenols and the bottle aperture size for acetone [22,23]. The collection of tsetse flies was conducted after 48 h of setting up the traps. A standard data sheet was used to record data such as date, location, trap type, trap number, vegetation type, number of flies caught, species, feed status, and sex of the fly. Flies were sorted using morphological identification based on species by looking at color, number of dark tarsal segments, size, wing venation, presence of bristles, and antennae shape, among other things [24]. Sex was identified based on the presence or absence of hypogium. Flies were then stored in 50 mL Falcon tubes containing 70% ethanol and shipped to the laboratory at Kenya Agricultural and Livestock Research Organization (KALRO) for further molecular analysis.

### 2.2. Molecular Experiments

For molecular analysis to detect infected tsetse flies, the genetic diversity of the trypanosome species in infected tsetse flies and the prevalence of the drug-resistant species in the flies, tsetse flies belonging to the same species from the same transect and the same county were grouped together in a pool of 20 flies each and dried off to remove excess ethanol. The flies were then crushed using a mortar and pestle, and each motor and pestle was then cleaned using bleach (sodium hypochlorite), followed by ethanol of 70%, and later distilled water before they could be used again for crushing. Subsequently, DNA was extracted from each pool of tsetse flies. DNA extraction from the pools of flies was performed using blood and tissue Qiagen DNA extraction kit according to manufacturer’s instructions. The DNA was then preserved in a fridge at −20 °C. For conventional polymerase chain reaction (PCR), primers for nested rRNA genes were sourced from Macrogen in Germany.

Nested PCR was applied using primers ITS1-ITS2 and ITS3-ITS4. These primers bind to 18S, ITS1, 5.8S, ITS2, and the 28S regions of rDNA. First, ITS1 and ITS2 primers were used, where PCR was conducted in a 25 μL reaction consisting, 1X buffer (Bioline, London, UK), 1 μM F, and 1 μM R primers, 12.6 μL DNase-free water, 0.8 U/μL MyTaq (Bioline, UK), and 5 μL of DNA. Later, ITS3 and ITS4 primers were used by adding 5 μL of PCR product for the ITS1 and ITS2 reaction to 20 μL of the PCR reaction mixtures with ITS3 and ITS4 primers minus the template DNA [25]. Electrophoresis was conducted in all the amplified PCR products using 2% agarose gel (Meridian Bioscience-Bio41026, Cincinnati, OH, USA), stained with GelRed (Biotium Hayward, Fremont, CA, USA), and viewed under Gel imager-UVITEC Cambridge.

All *T. brucei*-positive samples were subjected to PCR amplification of *TbAT1* transporter genes [26], while all *T. congolense*-positive samples were subjected to PCR amplification of *DMT*, *E6M61*, and *TcoAde2* [15,16,17]. Gel electrophoresis was then performed as explained above to view the bands. Polymerase chain reaction restriction fragment length polymorphism (PCR-RFLP) was used to detect *Trypanosoma* species resistant to diminazene aceturate [17,19]. A DNA fragment of the Trypanosoma adenosine transporter gene *Ade2*, which was positive with the PCR test, was purified using GeneJET PCR purification Kit (Thermo Scientific, Waltham, MA, USA) using the manufacturer’s instructions. A part (10 μL) of the purified PCR product from Ade2 gene was equally divided into two 5 μL and was digested with DpnII (recognition sequence ˆGATC), and the other 5 uL was digested with BclI (recognition sequence TˆGATCA) endonuclease, at 37 °C overnight. The digestion of the Ade2 gene with BclI would ideally yield fragments of 354–256–38 bp and 610–38 bp for resistant and susceptible strains, respectively. Meanwhile, the digestion of the Ade2 gene with DpnII would ideally give 400–248 bp for resistant strains and no digestion (648 bp) for sensitive strains. Digestion products were then electrophoresed on a 1.5% agarose gel. Another part (15–20 μL) of the purified PCR products from the *Ade2* gene was then submitted to Inqaba Biotec for direct sequencing by the Sanger method. All other PCR products that were positive for the trypanosomes and other transporter gene-positive samples were also purified using the GeneJET PCR purification Kit (Thermo Scientific) using the manufacturer’s instructions and later sent to Inqaba Biotec for direct Sanger sequencing.

### 2.3. Data Analysis

Entomological tsetse abundance and apparent density were expressed in terms of flies per trap per density (FTD). FTD was calculated using the formula FTD = ΣF/T X D, where ΣF represents the total number of flies captured, T the number of functional traps used to catch the flies, and D the number of days the traps were functional. To edit the generated sequences, the BioEdit program was used. Sequences were then blasted using Blastn in NCBI (https://www.ncbi.nlm.nih.gov/; accessed on 16 September 2025). Homologous nucleotide sequences between our query rRNA sequences and those related sequences in the GenBank were aligned using the Clustal W multiple alignment in the MEGA7 software version 7. Transporter gene sequences *TbAT1*, *DMT*, *E6M61*, and *TcoAde2*, together with homologous sequences and drug-sensitive sequences in the GenBank, were extracted and aligned using Clustal W in Mega7. The aligned sequences were then used to determine the best model for creating a phylogenetic tree in MEGA7. A maximum likelihood (ML) phylogenetic tree was then created from the aligned sequences using 1000 bootstraps [27]. Query sequences generated were also stored in GenBank.

A chi-square test for equality of proportions was used to compare (i) the apparent density of tsetse flies across counties and the apparent density of tsetse fly species within each county, (ii) the prevalence of trypanosome-positive cases across different counties, across various species of tsetse flies, and finally, (iii) across various tsetse fly species within each county.

## 3. Results

**Figure 2:**
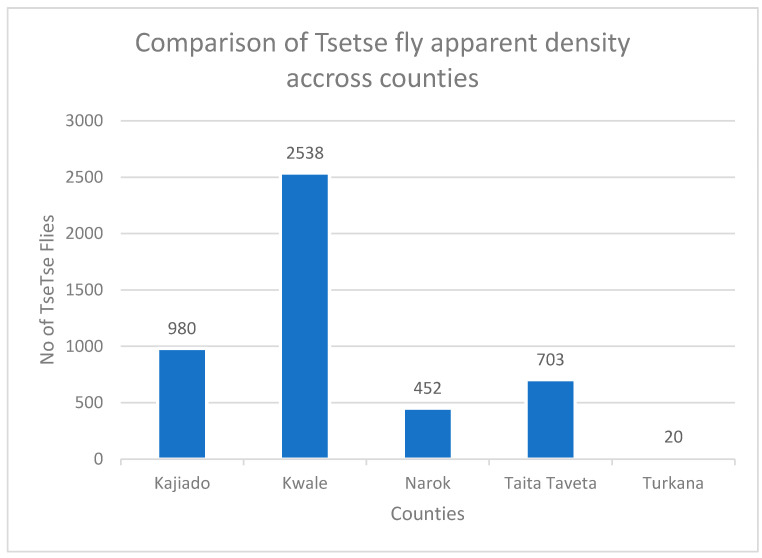
Graph showing comparison of tsetse fly apparent density across the five different counties.

A total of 4693 tsetse flies was collected. Generally, Kwale had the most tsetse flies collected from all five counties, n = 2538; hence, the flies trapped per trap per day (FTD) was 101.52. It was followed by Kajiado, n = 980, which had an FTD of 39.20; Taita-Taveta, n = 703, with an FTD of 46.86; Narok, n = 452, with an FTD of 14.44. Finally, 20 tsetse flies were captured in Turkana, with an FTD of 1.18—Figure 2. Hence, the apparent density of tsetse flies was statistically different across various counties (*p*-value < 0.001).

**Figure 3:**
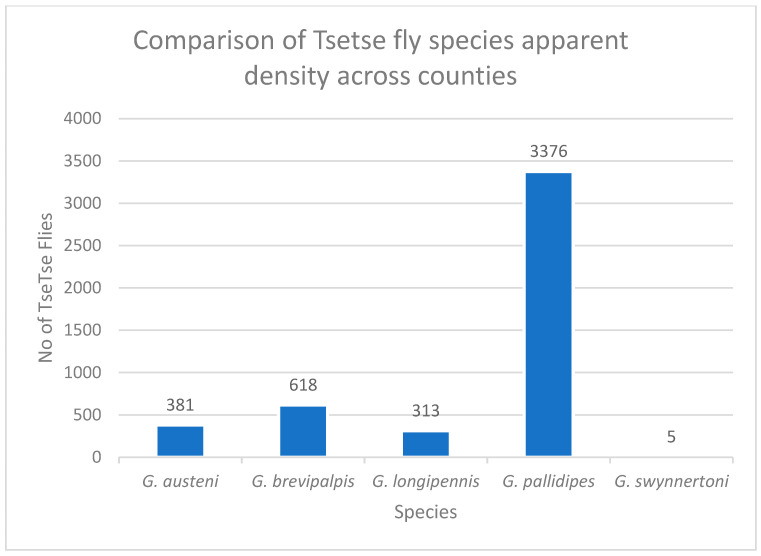
Graph showing comparison of tsetse fly species apparent density across the five different counties.

Five species of tsetse flies were collected in all five counties. *Glossina pallidipes* (n = 3376) was the most abundant species, followed by *G. brevipalpis* (n = 618), *G. austeni* (n = 381), *G. longipennis* (n = 313), and *G. swynnertoni* (n = 5)—Figure 3. Also, the apparent density of different tsetse fly species was statistically significant across the various counties (*p*-value < 0.001).

**Figure 4:**
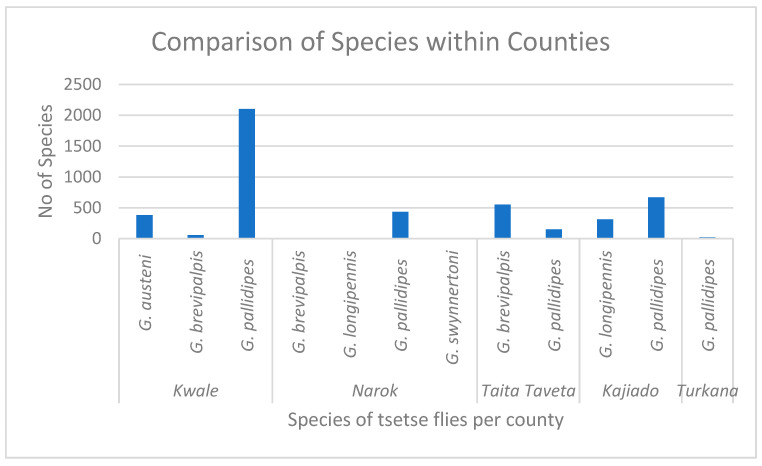
Graph showing comparison of tsetse flies’ species apparent density within the five different counties.

For tsetse fly species variation within each county, Kwale County had *Glossina pallidipes* (n = 2102), *G. austeni* (n = 381), *G. brevipalpis* (n = 55). In Taita-Taveta, two different species, *Glossina pallidipes* (n = 151) and *G. brevipalpis* (n = 552), were collected. In Kajiado County, two species, *Glossina pallidipes* (n = 668), *G. longipennis* (n = 312), were collected, and four different species, *G. pallidipes* (n = 435), *G. brevipalpis* (n = 11), *G. swynnertoni* (n = 5), and *G. longipennis* (n = 1), were collected in Narok County. Turkana had (n = 20) the *G. pallidipes* species collected—Figure 4. Thus, comparing the apparent density of different tsetse fly species within each county was statistically significant (*p*-value < 0.001).

**Figure 5:**
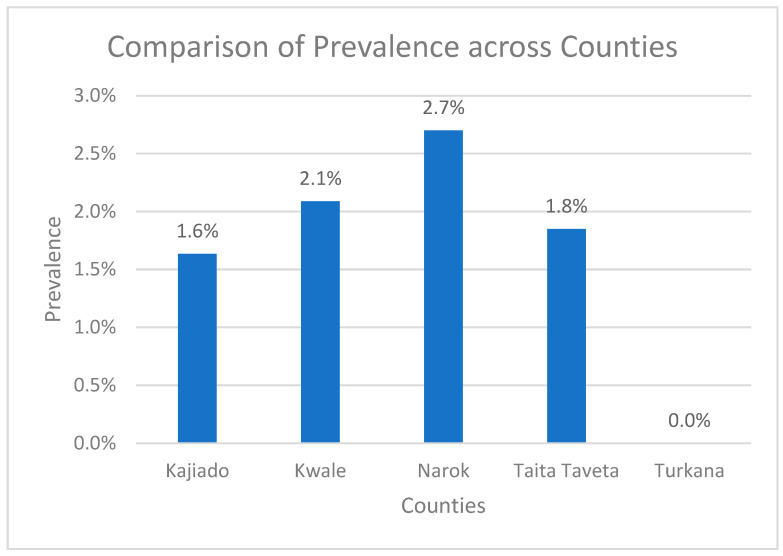
Graph showing comparison of trypanosome prevalence in tsetse flies across five different counties.

The expected band sizes were 611 bp for *T. vivax*, 1207–1224 bp for *T. brucei*, 1422 bp for *T. congolense* Kilifi, 1513 bp *for T. congolense* Forest, 1413 bp for *T. congolense* Savannah, 954 bp for *T. congolense* Tsavo, 850 bp for *T. simiae*, and 988 bp for *T. theileri* [25]. The molecular prevalence of trypanosome infections in tsetse flies was determined using nested ITS1-ITS2 and ITS3-ITS4 conventional PCR. Overall, 2.0% (93/4693) of the flies tested positive for trypanosome infections. In Narok County, the overall prevalence was 2.7% (12/452). Among the species, *G. pallidipes* had infected 11/435 flies (2.53%), and *G. brevipalpis* had 1/11 infections (9.09%). No infections were detected in *G. swynnertoni* (0/5) or *G. longipennis* (0/10). In Kwale County, the prevalence was 2.1% (53/2538). Here, *G. pallidipes* had 44/2102 infections (2.10%), *G. austeni* had 8/381 infections (2.10%), and *G. brevipalpis* had 1/55 infections (1.82%). In Taita-Taveta County, the prevalence was 2.0% (13/703). This included 11/552 infections in *G. brevipalpis* (2.00%) and 2/151 infections in *G. pallidipes* (1.33%). In Kajiado County, the overall prevalence was 1.6% (16/980). *G. pallidipes* recorded 13/668 infections (2.0%), while *G. longipennis* had 3/312 infections (1.00%). In Turkana County, no infections were detected. The prevalence was 0.0% (0/20) among *G. pallidipes*—Figure 5. However, there was no statistically significant difference in prevalent proportions across counties (*p*-value = 0.7304).

**Figure 6:**
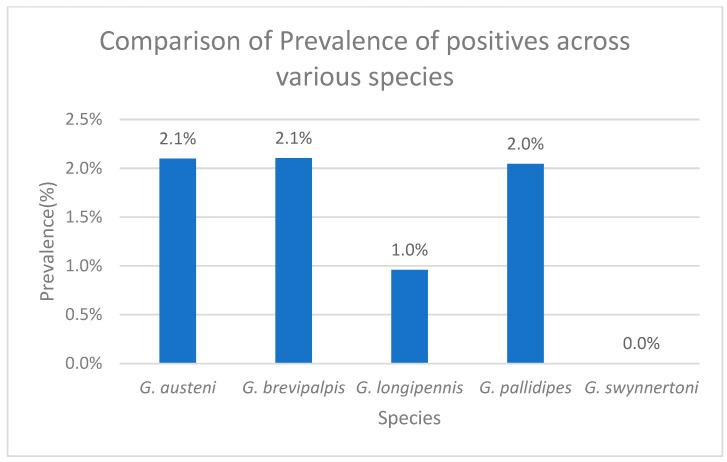
Graph showing comparison of trypanosome prevalence in tsetse flies across different tsetse species.

For the molecular comparison of trypanosome infection prevalence across different tsetse species, *G. brevipalpis* showed a prevalence of 2.10% (13/618), *G. austeni* 2.10% (8/381), *G. pallidipes* 2.04% (69/3376), *G. longipennis* 1.00% (3/313), and *G. swynnertoni* had no infections detected (0/5)—Figure 6.

**Figure 7:**
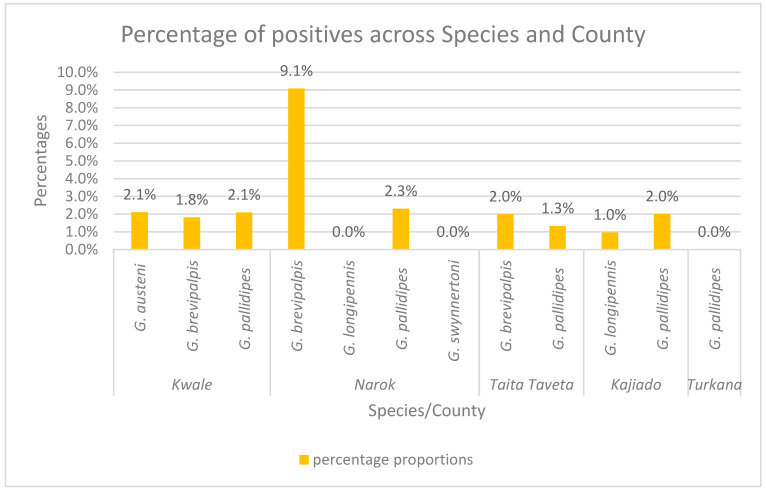
A graph showing comparison of trypanosome prevalence across various tsetse fly species across different counties.

In counties like Taita-Taveta, there were more *G. brevipalpis* captured (n = 552) than *G. pallidipes* (n = 151), and the prevalence of infections was higher in *G. brevipalpis* (2.0%) than in *G. pallidipes* (1.33%). In Kwale, *G. autseni* and *G. pallidipes* had the same prevalence of infections at 2.1% each, while in Narok, *G. brevipalpis* had a higher prevalence of infection at 9.1% than *G. pallidipes* at 2.3%—Figure 7.

Generally, we can say that some of the tsetse species that have more potential of carrying trypanosome infections from the five AAT endemic counties are *G. brevipalpis*, *G. autseni*, and *G. pallidipes*. In contrast, *G. longipennis*, followed by *G. swynnertoni*, has the least potential of carrying trypanosome infections. Distribution of the various trypanosome species detected in the tsetse fly species collected from the different counties is as shown below—Table 1. *T. congolense* was the most abundant species detected n = 29, followed by *T. brucei* n = 26, *T. vivax* n = 18, mixed *Tv + Tb* and mixed *Tc + Tb* (each n = 8), and mixed *Tv + Tc* n = 4 and mixed *Tv + Tb + Tc* n = 1—Table 1**.** *T. congolense* was mostly detected in Kwale County, while *T. brucei* was mostly detected In Kajiado and Kwale County, and finally, *T. vivax* and mixed *Tc + Tb* were also mostly detected in Kwale County.

### 3.1. Sequence Identification

For sequences generated from the identified infected tsetse flies, sizes were between 340 and 584 bp. For percentage identity, 90–100% was used to identify homologous sequences to our query sequences in GenBank. Four sequences identified as *T. vivax* from *G. pallidipes* collected in Kwale County were assigned the accession numbers PX051717, PX051722, PX051723, and PX051725. Sequence PX051717 showed a high similarity of 99.70% to *T. vivax* from Mozambique and 95.00–99.41% similarity to isolates from East Africa and Côte d’Ivoire. Sequence PX051722 showed a 97.56% similarity to *T. vivax* from Mozambique and 96.06–96.88% similarity to those from East Africa. Sequence PX051723 had a 98.97% similarity to *T. vivax* from Mozambique and 97.16–98.00% similarity to species from East Africa. Finally, sequence PX051725 showed a high similarity of 99.42–99.71% to *T. vivax* from both East Africa and Mozambique. Three *T. brucei* in *G. autseni* and *G. pallidipes* tsetse flies from Kwale and Taita-Taveta counties were identified as sequences for two fungal species, *Cutaneotrichosporon debeurmannianum* and *Gloeophyllum trabeum*, and these had 96.98–98.35% similarity to other related fungal species in GenBank, and were deposited in GenBank with accession numbers PX051716, PX051720, and PX051724.

### 3.2. Genetic Diversity Among Trypanosome Species in Tsetse Flies

When genetic comparisons were made between our query sequences and related species in GenBank, the four *T. vivax* sequences (accession numbers PX051722, PX051723, PX051717, and PX051725) showed 1530 nucleotides matching those in GenBank, with an overall similarity of 98.46%. A total of six deletions, two insertions, and fifteen substitutions were identified.

### 3.3. Phylogenetic Tree of Trypanosomes in Tsetse Flies Based on Amplified Partial 28S and Partial 18S, ITS1, 5.8S, ITS2 rRNA Genes

**Figure 8:**
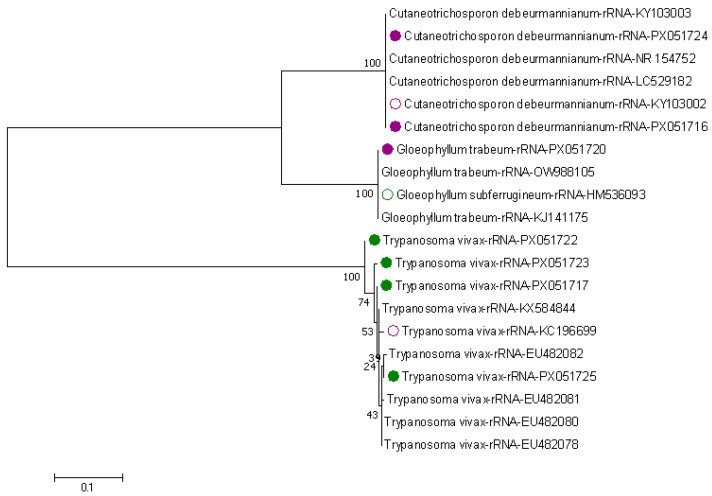
Maximum likelihood phylogenetic tree based on partial 18S, ITS1, 5.8S, ITS2, and the 28S regions of rRNA. Replicates of 1000 bootstraps were used to create the tree using Kimura-2 parameter model in MEGA7. The numbers on each branch are the bootstrap code value percentages. The species highlighted in green are our query *T. vivax* sequences, while those highlighted in pink are the fungus sequences identified from our study. The rest of the species not highlighted are homologous sequences from GenBank.

Upon applying the Kimura 2-parameter model and using 1000 bootstraps to form a maximum likelihood (ML) phylogenetic tree, the following tree was formed. Our query sequences are highlighted in green and purple—Figure 8. Our four query *T. vivax* sequences formed two distinct groups. Three of our *T. vivax* sequences with accessions PX051722, PX051723, and PX051717 were closely related to each other with a bootstrap code value of 100%, and they were also closely related to *T. vivax* from Mozambique with a bootstrap code value of 74%. The other *T. vivax* sequence with accession PX051725 had a low bootstrap value of (24–39%), and hence was not reliable in determining the phylogenetic relationship with other species. Sequences highlighted in purple—PX051724, PX051716, and PX051720—were identified as two fungus species *Cutaneotrichosporon debeurmannianum* and *Gloeophyllum trabeum*, and these had 100% bootstrap values, and were closely related to other similar fungus species directly submitted to GenBank.

### 3.4. Transporter Genes Detection and Sequence Comparisons

**Figure 9:**
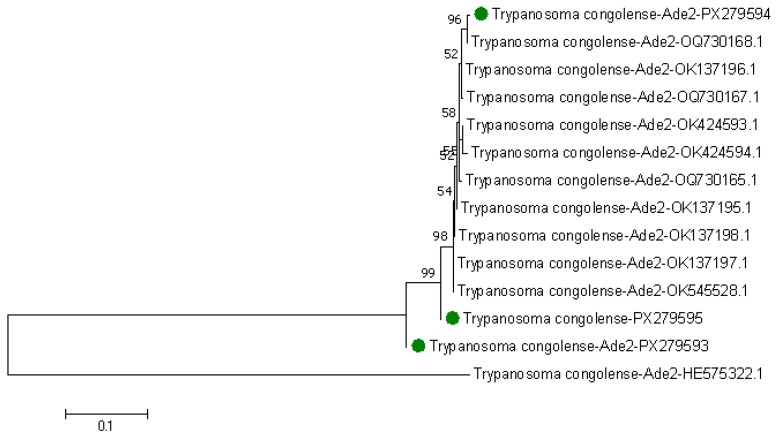
Maximum likelihood phylogenetic tree based on *Ade2* genes in *T. congolense* species. Replicates of 1000 bootstraps were used to create the tree using Tamura-3 parameter model in MEGA7. The numbers on each branch are the bootstrap code value percentages. The sequences highlighted in green are our query *T. congolense Ade2* gene sequences. The rest of the sequences not highlighted are homologous *Ade2* sequences from GenBank.

The expected band sizes were 1600 bp for *TbAT1*, 800 bp for *DMT*, 381–383 for *E6M61*, and 648 bp for *TcoAde2* genes. *Ade2* genes were detected in 11 *T. congolense* species (n = 10 in Kwale County and n = 1 in Taita-Taveta County) in n = 8 *G. pallidipes*, n = 2 *G. autseni*, and n = 1 *G. brevipalpis* tsetse species. Three of the *Ade2* sequences were reamplified using Sanger sequencing, and these were from Kwale County, which were later deposited in GenBank with accession numbers PX279594, PX279595, and PX279593. These sequences had (96.82–99.66%) percentage identity similarity to other *T. congolense* from the Ruma, Kamato, Gendo, and KETRI regions of Kenya. A maximum likelihood phylogenetic tree was created, and our query sequences are highlighted in green—Figure 9. One of the query sequences, PX279594, had a 96% bootstrap value in relation to *T. congolense* from Ruma in Kenya with accession OQ730168.1. Other query sequences, PX279595 and PX279593, were more closely related to *T. congolense* from Gendo and KETRI. However, our three query sequences were distantly related to a drug-sensitive species from GenBank with accession HE575322.1. For genetic variations, our three query sequences had 1275 nucleotides similar to those in GenBank, corresponding to 89.29% similarity, 122 deletions, and 31 substitutions.

**Figure 10:**
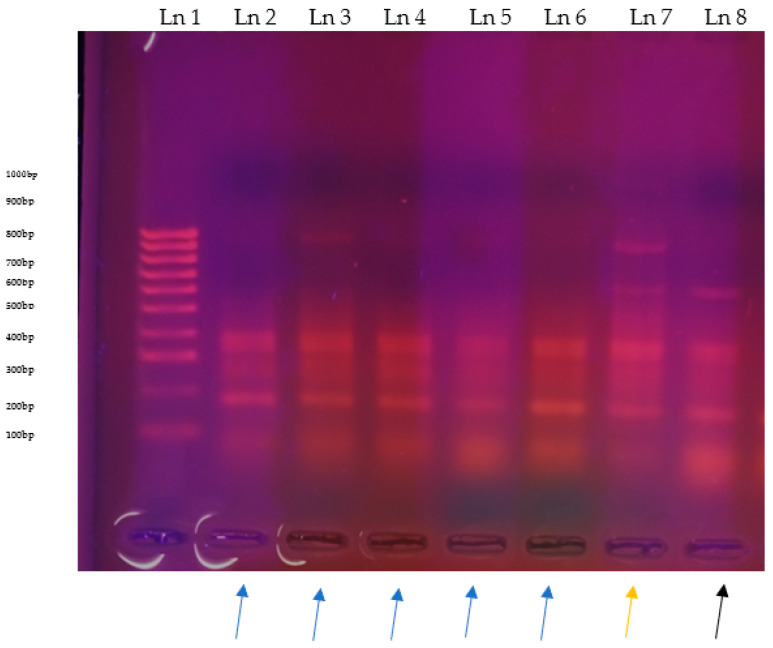
RFLP digestions of *Ade2* gel images: Ln 1, 100 bp ladder; Ln 2, 3, 4, 5, 6, samples with two bands 248–400 bp pointed with a blue arrow at the bottom; Ln 7, sample with four bands 248–400 bp, 648 bp and 800 bp pointed with a yellow arrow, Ln 8, samples with three bands 248–400 bp and 648 bp pointed with a black arrow at the bottom.

For RFLP, digestions of *Ade2* PCR products from Kwale using DpnII showed five strains of *T. congolense* with three bands, 248–400 bp and 648 bp (two bands associated with resistant strains and one band with sensitive strain), while five strains gave two bands, 248–400 bp (all associated with resistant strains). As for DpnII, digestions from the Taita-Taveta isolate also had three bands, 248–400 bp, and 648 bp, following similar traits as the ones in Kwale County—Figure 10. Digestion using the BcII enzyme showed three isolates of *T. congolense* in tsetse flies from Kwale County, having three bands of 38–256 bp and 354 bp (associated with drug resistant isolates), six isolates (five from Kwale and one from Taita-Taveta) had two bands, either 256 and 354 bp or 256 and 38 bp (bands associated with drug resistance in the isolates with only one band 38 bp missing).

**Figure 11:**
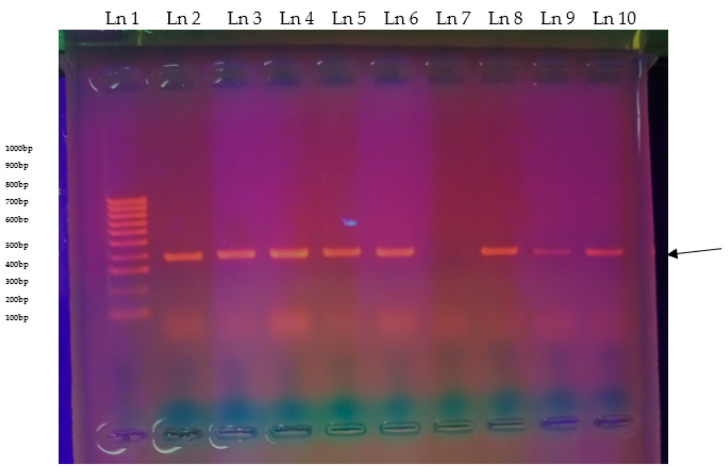
*E6M6* gel images, gel 1a: Ln 1, 100 bp ladder; Ln 2, 3, 4, 5, 6, 8, 9, 10, 383 bp bands pointed with a black arrow on the right; Ln 7, negative band.

Eleven *E6M6* transporter genes were detected in the *T. congolense* (n = 3 in Taita taveta and n = 8 in Kwale) identified in n = 8 *G. pallidipes*, n = 2 in *G. autseni*, and n = 1 in *G. brevipalpis* tsetse flies—Figure 11.

**Figure 12:**
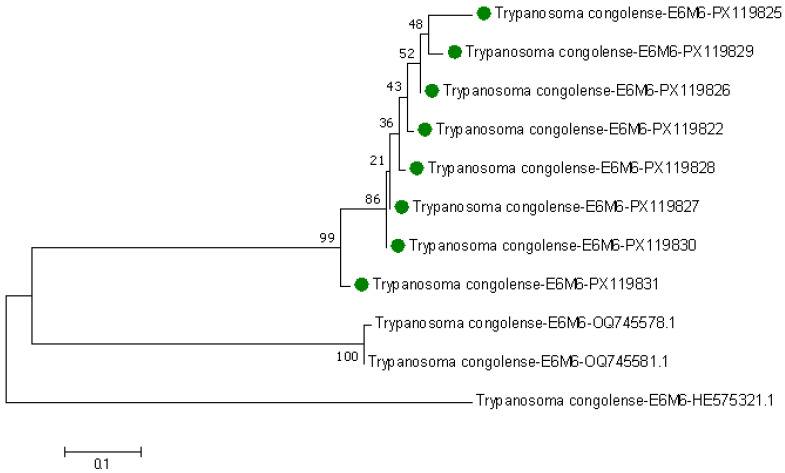
Maximum likelihood phylogenetic tree based on *E6M6* genes in *T. congolense* species. Replicates of 1000 bootstraps were used to create the tree using Tamura-3 parameter model in MEGA7. The numbers on each branch are the bootstrap code value percentages. The sequences highlighted in green are our query *E6M6* gene sequences. The rest of the sequences not highlighted are homologous *E6M6* sequences from GenBank.

Eight *E6M6* sequences were determined, and these were from Kwale, Taita-Taveta, and Kajiado and were later deposited in GenBank with accession numbers PX119822 (Kwale), PX119825 (Kwale), PX119826 (Kwale), PX119827 (Kwale), PX119828 (Taita), PX119829 (Kajiado), PX119830 (Kwale), and PX119831 (Kwale). These sequences had (85.56–99.70%) similarity to other similar sequences in GenBank from the Ruma region of Kenya. The sequences had a total of 2172 nucleotides that were similar to other homologous sequences in GenBank, hence having a percentage similarity of 85.02%, 300 deletions, 10 insertions, and 67 substitutions. For phylogenetic trees created using maximum likelihood, our query sequences are highlighted in green—Figure 12. Two of our sequences with accession numbers PX119827 and PX119830 were closely related to each other and had a bootstrap value of 86%. The other query sequence PX119831 was more closely related to our two sequences, PX119827 and PX119830, than to those from the GenBank OQ745578.1 and OQ745881.1 from the Ruma region in Kenya. However, these sequences were distantly related to sequence HE575322.1, a drug-sensitive species from GenBank.

On the other hand, *DMT* genes could not be detected in any of our *T. congolense* species. *TbAT/P2* transporter genes could also not be detected in our *T. brucei* species.

## 4. Discussion

This study investigated potential tsetse fly vectors of trypanosomes, prevalence, and genetic diversity of trypanosomes associated with drug resistance in tsetse species from five endemic counties of Kenya. The apparent density of flies captured varied in different tsetse fly species from the different counties. This is expected, as different tsetse flies thrive well in different ecological zones. The palpalis group, e.g., *Glossina tachinoides*, thrives well in humid environments, riverine vegetations, gallery forests, and regions close to large water bodies; the fusca group thrives well in closed-canopy forests; and the mortisan group thrives well in wood grasslands and savannah woodlands [28]. Also, the varying seasonal changes can affect the apparent density of flies collected; in our case, Turkana County had the lowest apparent density of tsetse flies, as the collection of flies was conducted during the beginning of the dry season when fly populations are normally considered to be low. Similar reports have been given in a different study in Kenya, from the Lambwe region [12]. Generally, we can say the tsetse fly species with the most apparent density in these five endemic regions of Kenya is *G. pallidipes*, followed by *G. brevipalpis*, *G. autseni*, *G. longipennis*, and *G. swynnertoni.* Similar reports have been published in Kenya by Ngari [1]. However, for our study, we could not detect *G. fuscipes fuscipes*, as our survey did not cover areas like Western Kenya Busia County, where this species of tsetse fly is known to be prevalent [29]. As for the potential tsetse fly vectors posing a risk of transmitting trypanosome infections, we can say this varies as per the different AAT endemic counties. In counties like Taita-Taveta, there were more *G. brevipalpis* than *G. pallidipes* captured, and the prevalence of trypanosome infections was higher in *G. brevipalpis* than in *G. pallidipes*. In Kwale County, there were more *G. pallidipes* than *G. autseni* and *G. brevipalpis* captured. However, *G. autseni* and *G. pallidipes* had the same prevalence of infections, showing these two tsetse species have similar potential of causing infections in Kwale County. In Narok, *G. brevipalpis* had a higher prevalence of infection than *G. pallidipes*, though the apparent density of *G. pallidipes* was higher than *G. brevipalpis*. Finally, in Kajiado County, the apparent density for *G. pallidipes* was higher than for *G. longipennis*, and the prevalence of trypanosome infection was also higher in *G. pallidipes*, showing that *G. pallidipes* has better chances of transmitting infections than *G. longipennis* in Kajiado. However, other factors, such as the feeding capacity of the tsetse flies, should also be considered, as this will also determine the probability of tsetse flies causing infections.

As for sequence results, based on the amplified rRNA genes, the level of similarity of our identified *T. vivax* to other similar species showed that there is less genetic diversity in our sequences. This can be explained by the fact that we only had a few sequences generated for *T. vivax*, which may affect the number of homologous sequences to our query sequences identified in GenBank, thus affecting the level of genetic diversity detected. Similar suggestions have been given in a different study when the number of sequences compared were high then the number of insertions and deletions increased, increasing the genetic diversity and error rates within the species [30,31]. When considering homologous sequences based on the percentage identity of our query for *T. vivax* sequences, our *T. vivax* species were closely related to species from Mozambique, Cote d’Ivoire, and East African species. Moreover, our *T. vivax* sequences formed two distinct groups in the maximum likelihood phylogenetic tree, which were related to species from Mozambique. This can be attributed to some level of genetic diversity within our sequences, associating them with other sequences from Southeast, West, and East Africa. Similar reports of *T. vivax* having such genetic diversity have been reported in studies from Cote d’Ivoire [10,32]. This can also be explained by more studies on the genetics of *T. vivax* having been conducted in these regions of Africa, hence having more information compared to other countries in Sub-Saharan Africa. Some *T. brucei* species detected through PCR were identified to be two different species of fungus. This can be attributed to the fact that the amplified rRNA region within the *T. brucei* species shares some conserved regions with these fungal species.

As for the drug resistance tests using restriction fragment length polymorphism (RFLP), there were *T. congolense* strains from Kwale and Taita-Taveta County traits associated with drug resistance. Our suggestion for this is that the poor use of trypanocidal drugs within Kwale County may potentiate the emergence of resistant isolates. Similar reports from Kwale County have been given, showing inappropriate use of drugs and suggestions of drug resistance from the region [21]. A different study on the prevalence of trypanosome associated with drug resistance in Kwale County and Shimba Hills did not detect any *T. brucei* associated with drug resistance [11]. In our study, no *T. brucei* transporter genes were detected in tsetse flies from KwaIe County. As for Taita-Taveta County, no known study has been conducted to report cases of trypanocidal drug resistance in the region. However, a recent county report from Taita-Taveta mentioned AAT as one of the common diseases affecting livestock in the region [33]. In our study, there were *T. congolense* strains, which had mixed bands for resistant and sensitive strains in both Kwale and Taita-Taveta. These can be associated with mixed *T. congolense* infections, with some strains being resistant and others being sensitive. For strains that had two bands instead of three bands associated with drug-resistant strains, we can say that these were strains that did not fully digest during the incubation period during the RFLP experiment, to give the three bands associated with drug resistance. Hence, for future studies, more incubation time should be given for RFLP to give better results.

When considering sequences for our identified transporter genes *TcoAde2* and *E6M6* in *T. congolense*, these genes had 82.29% and 85.02% similarity, respectively, to other sequences in the GenBank. However, with the phylogenetic trees created, *TcoAde2* transporter genes had more genetic diversity than *E6M6* transporter genes. This is because the *TcoAde2* sequences of our query sequences were coming from one region, Kwale, but were related to sequences from other parts of Kenya, such as Ruma, Gendo, KETRI, and Kamato, but for *E6M6* transporter genes, our query sequences were more closely related to each other than to other sequences from other regions. Similar reports of genetic diversity observed in the transporter genes have been documented in a different study in Kenya by Okello [12]. Since our transporter genes were distantly related to a drug-sensitive species in GenBank, it could mean that they could have developed some mutations that affect their drug uptake. Our RFLP experiments have shown that indeed we may be having some *T. congolense* isolates from Kwale and Taita-Taveta that are associated with drug resistance from our study. Hence, this raises questions as to whether more studies on drug resistance need to be conducted within and beyond the regions.

## 5. Conclusions

This study reveals that different tsetse fly species are present in Kenya, with their apparent densities varying across regions. Some tsetse species exhibit a higher risk of transmitting trypanosomes, suggesting they are key drivers of trypanosomiasis in various geographical areas. *Trypanosoma* species prevalence in tsetse flies varies, with certain species being more common in specific endemic regions. Notably, strains of *T. congolense* associated with drug resistance were found in the Kwale and Taita-Taveta regions of Kenya, which may hinder efforts to eliminate AAT. Furthermore, our study identified genetic diversity in the transporter genes *TcoAde2* and *E6M6* in *T. congolense* species from the Kwale region. These findings highlight the need for targeted control of high-risk tsetse species and drug-resistant trypanosomes in Kenya.

## Figures and Tables

**Table 1:** Interspecies distribution of various tsetse fly species with different trypanosome species detected in the tsetse flies across different counties.

Narok	*Tb*	*Tv*	*Tc*	Mixed *Tv + Tb*	Mixed Tv + Tc	Mixed *Tc + Tb*	Mixed *Tv + Tc + Tb*	Total Infections	Total Tsetse Flies
*G. pallidipes*	4	4	0	3	0	0	0	11	435
*G. brevipalpis*	0	0	0	1	0	0	0	1	11
*G. swynnertoni*	0	0	0	0	0	0	0	0	5
*G. longipennis*	0	0	0	0	0	0	0	0	1
Total	4	4	0	4	0	0	0	12	452
Kwale	*Tb*	*Tv*	*Tc*	Mixed *Tv + Tb*	Mixed *Tv + Tc*	Mixed *Tc + Tb*	Mixed *Tv + Tc + Tb*	Total infections	Total tsetse flies
*G. pallidipes*	6	6	21	1	3	7	0	44	2102
*G. autseni*	2	2	4	0	0	0	0	8	381
*G. brevipalpis*	0	0	0	0	1	0	0	1	55
Total	8	8	25	1	4	7	0	53	2538
Taita-Taveta	*Tb*	*Tv*	*Tc*	Mixed *Tv + Tb*	Mixed *Tv + Tc*	Mixed *Tc + Tb*	Mixed *Tv + Tc + Tb*	Total infections	Total tsetse flies
*G. pallidipes*	0	0	2	0	0	0	0	2	151
*G. brevipalpis*	4	4	1	1	0	1	0	11	552
Total	4	4	3	1	0	1	0	13	703
Kajiado	*Tb*	*Tv*	*Tc*	Mixed *Tv + Tb*	Mixed *Tv + Tc*	Mixed *Tc + Tb*	Mixed *Tv + Tc + Tb*	Total infections	Total tsetse flies
*G. pallidipes*	9	2	1	1	0	0	0	13	668
*G. longipennis*	1	0	0	1	0	0	1	3	312
Total	10	2	1	2	0	0	1	15	980
Turkana	*Tb*	*Tv*	*Tc*	Mixed *Tv + Tb*	Mixed *Tv + Tc*	Mixed *Tc + Tb*	Mixed *Tv + Tc + Tb*	Total infections	Total tsetse flies
*G. pallidipes*	0	0	0	0	0	0	0	0	20
Total	0	0	0	0	0	0	0	0	20
Overall total	26	18	29	8	4	8	1		

Key: *Tb: T. brucei*, *Tv: T. vivax*, *Tc: T. congolense*, mixed *Tv + Tb*: mixed *T. vivax + T. brucei*, mixed *Tv + Tc*: mixed *T. vivax + T. congolense*, mixed *Tc + Tb*: mixed *T. congolense + T. brucei*, mixed *Tv + Tc + Tb*: mixed *T. vivax*, *T. congolense* + *T. brucei*.

## Data Availability

All sequence data associated with this study are deposited in GenBank.

## Abbreviations

KENTTEC	Kenya Tsetse and Trypanosomiasis Eradication Council
AAT	African animal trypanosomiasis
rRNA	ribosomal RNA gene
ITS1	Internal transcribed spacer 1 region
DMT	drug metabolite transporter
RFLP	restriction fragment length polymorphisms
TbAT/P2	*Trypanosome brucei* adenosine transporter gene
E6M6	ATP-binding cassette (ABC) transporter gene
TcoAde2	*Trypanosoma congolense* adenosine transporter gene
